# The Human Placental Amniotic Membrane Mesenchymal-Stromal-Cell-Derived Conditioned Medium Inhibits Growth and Promotes Apoptosis of Human Cholangiocarcinoma Cells In Vitro and In Vivo by Suppressing IL-6/JAK2/STAT3 Signaling

**DOI:** 10.3390/cells12242788

**Published:** 2023-12-07

**Authors:** Tanachapa Jantalika, Sirikul Manochantr, Pakpoom Kheolamai, Duangrat Tantikanlayaporn, Nattaya Thongsepee, Naree Warnnissorn, Weerachai Saijuntha, Somchai Pinlaor, Chairat Tantrawatpan

**Affiliations:** 1 Division of Cell Biology, Department of Preclinical Sciences, Faculty of Medicine, Thammasat University, Pathum Thani 12120, Thailand; tanachapa.jantalika@gmail.com (T.J.); bsirikul@gmail.com (S.M.); pkheolamai@me.com (P.K.); dkanlayaporn@gmail.com (D.T.); 2 Center of Excellence in Stem Cell Research and Innovations, Thammasat University, Pathum Thani 12120, Thailand; 3 Division of Physiology, Department of Preclinical Sciences, Faculty of Medicine, Thammasat University, Pathum Thani 12120, Thailand; nattayat@tu.ac.th; 4 Research Unit in Nutraceuticals and Food Safety, Thammasat University, Pathum Thani 12120, Thailand; 5 Department of Pathology, Faculty of Medicine, Thammasat University, Pathum Thani 12120, Thailand; nareeaw@gmail.com; 6 Faculty of Medicine, Mahasarakham University, Maha Sarakham 44000, Thailand; weerachai.s@msu.ac.th; 7 Department of Parasitology, Faculty of Medicine, Khon Kaen University, Khon Kaen 40002, Thailand; psomec@kku.ac.th

**Keywords:** mesenchymal stromal cells (MSCs), cholangiocarcinoma, conditioned medium, apoptosis, proliferation, placenta

## Abstract

Mesenchymal stromal cells (MSCs) have recently been shown to play an important role in the growth and progression of many solid tumors, including cholangiocarcinoma (CCA). The human placental amniotic membrane (hPAM) is one of the most favorable sources of MSCs due to its availability and non-invasive harvesting procedure. However, the role of human placental amniotic membrane mesenchymal stromal cells (hPAMSCs) in the growth and progression of human CCA has not yet been determined. This study investigates the effects of conditioned medium derived from hPAMSCs (PA-CM) on the properties of three human CCA cell lines and explores possible mechanisms of action. Varying concentrations of PA-CM were used to treat CCA cells to determine their effects on the proliferation and apoptosis of CCA cells. The results showed that PA-CM inhibited the proliferation and colony-forming capacity of KKU100, KKU213A, and KKU213B cells. PA-CM also promoted the apoptosis of these CCA cells by causing the loss of mitochondrial membrane potential. Western Blotting confirmed that PA-CM induced CCA cell apoptosis by increasing the levels of the Bax/Bcl-2 ratio, cleaved caspase 3, and cleaved PARP, possibly by inhibiting the IL-6/JAK2/STAT3 signaling pathway. Moreover, our in vivo study also confirmed the suppressive effect of hPAMSCs on CCA cells by showing that PA-CM reduced tumor volume in nude mice transplanted with human CCA cells. Taken together, our results demonstrate that PA-CM has potent tumor-suppressive effects on human CCA cells and could potentially be used in combination with chemotherapy to develop a more effective treatment for CCA patients.

## 1. Introduction

Cholangiocarcinoma (CCA) is an aggressive malignancy that originates from damaged epithelial cells at various sites within the intrahepatic or extrahepatic bile ducts [1]. Recently, the incidence of CCA has increased worldwide [2]; the lack of effective treatments [3,4] leads to a high mortality rate in CCA patients, with a 5-year overall survival rate of only 18% [5]. Therefore, it is imperative to find alternative therapeutic approaches to improve the effectiveness of CCA treatment.

Mesenchymal stromal cells (MSCs) are multipotent stem/stromal cells that have self-renewal and multilineage differentiation capacities [6]. Previous findings indicate that MSCs can migrate to the tumor, a property called ‘tumor tropism’, and secrete a combination of soluble factors, which affect several aspects of cancer cell properties by activating various cell signaling pathways [7]. According to several studies, MSC-secreted cytokines influence the IL-6/JAK2/STAT3 signaling pathway in cancer cells, which, in turn, can either stimulate or suppress tumor growth or trigger cancer cell apoptosis [8,9,10]. All of these effects make MSCs a very attractive target for the development of new cancer therapies. Although several studies have shown that MSC-derived soluble factors affect the growth, survival, and progression of various solid tumors, including ovarian cancer [11], hepatocellular carcinoma [12], and breast cancer [13], the effects of MSC-derived soluble factors on the growth and progression of CCA have not yet been fully characterized.

Human MSCs (hMSCs) have been successfully isolated from adipose tissue [14] and various gestational tissues [15], as well as bone marrow [16]. Among these sources, human placental amniotic membrane mesenchymal stromal cells (hPAMSCs) are considered one of the most favorable sources of MSCs for clinical application due to the easy and painless collection procedure, the high number of isolated hMSCs, the high level of cytokine production, and the strong tumor tropism [17,18,19].

Therefore, the present study aims to investigate the effects of conditioned medium derived from hPAMSCs (PA-CM) on the proliferation and apoptosis of three human CCA cell lines, KKU100, KKU213A, and KKU213B, in both in vitro and in vivo models. We also studied the molecular mechanism and signaling pathways that mediate the effects of hPAMSC-derived soluble factors on CCA cells. A more precise understanding of how hPAMSCs affect CCA growth and progression, as well as the mechanism that mediates these effects, could have substantial clinical implications for the development of more effective CCA treatments.

## 2. Materials and Methods

### 2.1. Isolation and Characterization of hPAMSCs

The placental tissues (*n* = 5) were obtained from full-term pregnant women after normal deliveries at the Thammasat University Hospital, Pathum Thani, Thailand. The mothers provided their informed written permission. The Human Ethics Committee of Thammasat University No. 1 (Faculty of Medicine; No. 258/2022) approved this study, which was carried out in accordance with the Declaration of Helsinki, the Belmont Report, and the ICH-GCP. Placental-amniotic-membrane-derived cells were prepared as follows. The placental amniotic membrane was washed with phosphate-buffered saline (PBS) and cut into small pieces. Subsequently, 0.5% trypsin EDTA was used to digest tissues for 2 h at 37 °C with shaking. After incubation, all tissues and cells were washed twice with PBS before culture with Dulbecco’s Modified Eagle Medium (DMEM; Gibco, Rockford, IL, USA) supplemented with 10% fetal bovine serum (FBS; Invitrogen, Waltham, MA, USA), 1% L-glutamine (Gibco, USA), and 1% penicillin/streptomycin (Gibco, USA) in a humidified atmosphere of 5% CO_2_ at 37 °C. 

The cells isolated at Passage Numbers 2–3 were identified as the primary culture of hPAMSCs by their fibroblast-like appearance, immunocytochemistry, and differentiation capacity. Flow cytometry analysis was used to identify cell surface antigens, comprising MSC markers CD73 (344004, BioLegend, San Diego, CA, USA), CD90 (328108, BioLegend, USA), and CD105 (560839, BD Bioscience, San Jose, CA, USA) and hematopoietic markers CD34 (343506, BioLegend, USA) and CD45 (304006, BioLegend, USA); IgG served as an isotype control. To examine the differentiation capacity, cells were cultured in adipogenic differentiation medium (DMEM containing 10% FBS, 2 mM L-glutamine (Gibco, USA), 100 U/mL penicillin, 100 µg/mL streptomycin (Gibco, USA), 0.5 mM isobutyl methylxanthine (Sigma-Aldrich, St. Louis, MO, USA), 1 µM dexamethasone (Sigma-Aldrich, USA), 10 µM insulin (Sigma-Aldrich), and 100 µM indomethacin (Sigma-Aldrich, USA)) and osteogenic differentiation medium (DMEM containing 10% FBS, 100 U/mL penicillin, 100 µg/mL streptomycin, 100 nM dexamethasone, 10 mM β-glycerophosphate (Sigma-Aldrich, USA), and 50 µg/mL ascorbic acid (Sigma-Aldrich, USA)). After 2–3 weeks of induction, the adipogenic and osteogenic differentiation potential of the isolated cells was determined by oil-red O and alizarin-red S staining, respectively.

### 2.2. Preparation of the Conditioned Medium

Human placental amniotic membrane mesenchymal stromal cells (hPAMSCs) were cultured to 80% confluence, subsequently washed twice with PBS, and further incubated in H-DMEM without FBS for 24 h. Conditioned medium derived from hPAMSCs (PA-CM) was collected, centrifuged at 1000× *g* for 5 min, and then filtered through a 0.22 µm filter. PA-CM was concentrated by lyophilizing at −52 °C, vacuum level 0.027 mBar for 72 h, and then kept at −80 °C. The freeze-dried PA-CM was reconstituted with DMEM to a 5X concentration and then added to the culture medium to provide final concentrations of 10%, 25%, 50%, and 75%, supplemented with 10% FBS.

### 2.3. Cell Lines and Culture

Three CCA cell lines (KKU100, KKU213A, and KKU213B), were cultured and expanded for use in all experiments. KKU100 was a low invasive cell line, derived from extrahepatic CCA with poor differentiation [20]. KKU213A was a highly invasive cell line with poor differentiation and was derived from intrahepatic CCA while KKU213B was a low invasive cell line and was derived from intrahepatic CCA with well differentiation [21]. KKU213A was cultured in H-DMEM supplemented with 10% FBS, 1% L-glutamine, and 1% antibiotic-antimycotic solution. Ham’s F-12 nutrient mixture medium (Gibco, USA), which included 10% FBS, 1% L-glutamine, and 1% antibiotic-antimycotic solution, was used to culture KKU100 and KKU213B cells in a humidified atmosphere of 5% CO_2_ at 37 °C.

### 2.4. MTT Assay

To determine the effect of PA-CM on the cell viability of CCA cell lines, an MTT assay was performed. Flat-bottom 96-well plates were seeded overnight with KKU100 (2.7 × 10^3^ cells/cm^2^), KKU213A (0.9 × 10^3^ cells/cm^2^), and KKU213B (0.9 × 10^3^ cells/cm^2^). A range of concentrations (0%, 10%, 25%, 50%, or 75%) of PA-CM, which included 10% FBS, were added to the CCA cell culture medium. Cell viability was detected every 24 h for 5 days by MTT assays, according to the manufacturer’s instructions. Briefly, 20 µL of MTT (5 mg/mL) was added to each well and incubated for 4 h. After incubation, the entire solution was discarded and 100 µL of dimethyl sulfoxide (DMSO) was added to solubilize the purple formazan crystals. Cell viability was measured by absorbance at 570 nm using a microplate reader. 

### 2.5. Colony Formation Assay

In total, 1000 CCA cells were seeded in six-well plates overnight. Subsequently, a final concentration of 0% (control), 10%, 25%, 50%, or 75% of PA-CM was added to the CCA cell culture medium, which was supplemented with 10% FBS. On day 6 for KKU213A and KKU213B and day 18 for KKU100 (approximately 6 times of cell division), the cells were washed with PBS, fixed in 10% formaldehyde for 15 min, and stained with 0.5% crystal violet solution for 20 min at room temperature. The number of colonies with more than 50 cells was counted using an inverted microscope with 40× magnification.

### 2.6. Flow Cytometry Analysis of Apoptosis

The annexin V/propidium iodide kit (BioLegend, USA) was used to determine the effect of PA-CM on the induction of the apoptosis of CCA cell lines. CCA cell lines (1 × 10^5^ cells/cm^2^) were seeded overnight and cultured with 0%, 50%, and 75% concentrations of PA-CM. All cells were harvested, washed, and resuspended at 12 h and 24 h in a binding buffer. Subsequently, cells were double stained with annexin V-fluorescein isothiocyanate (FITC) and propidium iodide (PI) solution and incubated for 15 min in the dark. The apoptotic cells were analyzed by flow cytometry.

To confirm the involvement of caspase activation in PA-CM-induced apoptosis in CCA cell lines, 20 µM Z-VAD(OMe)-FMK (pan-caspase inhibitor; Abcam, Cambridge, UK) was used to pretreat the CCA cells for 1 h, which were then treated with PA-CM at 0%, 50%, and 75% concentrations for 24 h. Flow cytometric analysis with annexin V–PI staining was performed.

### 2.7. Mitochondrial Membrane Potential (∆Ψm) Assay

The JC-1 staining assay kit (Abcam, UK) was used as an indicator of the mitochondrial membrane potential in the cells. The effect of PA-CM on mitochondrial membrane potential in CCA cells was examined by seeding CCA cell lines (1 × 10^5^ cells/cm^2^) overnight, which were cultured with PA-CM at 0%, 50%, and 75% concentrations for 12 h and 24 h. Carbonyl cyanide p-trifluoromethoxy phenylhydrazone (FCCP) was used to treat CCA cells at 100 μM for 4 h to serve as a positive control. All cells were stained with 10 μM JC-1 dye solution at 37 °C for 30 min and observed using a fluorescence microscope. Red fluorescence indicated JC-1 aggregation in normal mitochondria. When the membrane potential was reduced in an apoptosis cell, JC-1 was converted to a monomer with green fluorescence. A fluorescence plate reader was used for quantitative analysis at a signal strength of Ex475 nm/Em530 nm (green fluorescence) and Ex475 nm/Em590 nm (red fluorescence). A decrease in the red/green fluorescence ratio was an indication of mitochondrial depolarization. 

### 2.8. Caspase 3 Activity Assay

The activity of caspase 3 was evaluated using a caspase 3 colorimetric assay kit (Abcam, UK), according to the manufacturer’s instructions. In summary, CCA cell lines were seeded (1 × 10^5^ cells/cm^2^) overnight, treated with PA-CM (0%, 50%, and 75%) for 8 h, and, after incubation, they were lysed with 50 µL of chilled lysis buffer for 10 min and centrifuged at 10,000× *g* for 1 min at 4 °C to collect the supernatant. The protein sample was adjusted to 200 µg per 50 µL cell lysis buffer and transferred to a 96-well plate. For each sample, 50 µL of reaction buffer (containing 10 mM dithiothreitol) and 50 µL of caspase substrate (200 µM final concentration) were added and incubated at 37 °C for 2 h. A microplate reader at 405 nm was used to measure the activity of caspase 3, which was expressed as the optical density (OD).

### 2.9. Western Blot Analysis

CCA cells (1 × 10^5^ cells/cm^2^) were seeded overnight and cultured with PA-CM (0%, 50%, and 75%) for 8 h, whereupon the protein was collected. In total, 20 µM Z-VAD(OMe)-FMK (pan-caspase inhibitor) was used to pretreat CCA cells for 1 h, which were then treated with PA-CM (0% and 75%) for 8 h, to confirm the caspase apoptosis pathway mediated by PA-CM. Furthermore, CCA cells were pretreated with PA-CM (0% and 75%) for 8 h and stimulated with 100 ng/mL interleukin 6 (IL-6) (BioLegend, USA) for 30 min to determine whether PA-CM could inhibit the expression of IL-6-mediated JAK2/STAT3. After incubation, cells were collected and lysed with RIPA lysis buffer (Cell Signaling, Danvers, MA, USA), containing a protease inhibitor cocktail (Abcam, UK) on ice. Equal amounts of total protein were subjected to 12% SDS-PAGE and then electro-transferred onto nitrocellulose membranes. After blocking with 5% skimmed milk at room temperature for 1 h, blots were detected at 4 °C overnight with the following primary antibodies: B-cell lymphoma protein 2 (Bcl-2; #4223, Cell Signaling, USA), Bcl-2-associated X protein (Bax; #5023, Cell Signaling, USA), cleaved caspase 3 (#9664, Cell Signaling, USA), cleaved poly (ADP-ribose) polymerase (PARP; #5625, Cell Signaling, USA), signal transducer and activator of transcription 3 (STAT3; #12640, Cell Signaling, USA), phospho-STAT3 (p-STAT3; #9145, Cell Signaling, USA), Janus kinase 2 (JAK2; #3230, Cell Signaling, USA), and phospho-JAK2 (p-JAK2; #3776, Cell Signaling, USA). β-Actin (#66009-1-IG, Proteintech, Rosemont, IL, USA) acted as a loading control reference. Subsequently, the membrane was incubated at room temperature for 1 h with HRP-labeled mouse anti-rabbit (211-032-171, Jackson ImmunoResearch Laboratories, West Grove, PA, USA) or goat anti-mouse IgG (115-035-166, Jackson ImmunoResearch Laboratories, USA) at 1:10,000 dilution. An ECL system (Bio-Rad Laboratories, Inc., Hercules, CA, USA) was used to visualize specific proteins, which were quantified by an Amersham^TM^ Imager 600 (GE Healthcare, Tokyo, Japan).

### 2.10. In Vivo Assay

Six-week-old healthy male BALB/c-nu/nu mice with body weights between 18.5 and 22.0 g were purchased from Nomura Siam International Co., Ltd., Bangkok, Thailand. All the mice were housed in a room with a 12 h light:12 h dark cycle and provided with sterile food and water ad libitum. The temperature was controlled at 22 ± 2 °C in a barrier facility at the Laboratory Animal Center, Thammasat University. All experimental studies were carried out in accordance with the relevant ethical guidelines approved by the Institutional Animal Use and Care Committee of Thammasat University (approval number: 017/2021). A total of sixteen mice were included and randomly divided into four groups, with each group having four mice. To implant CCA tumors, 2 × 10^6^ KKU213B cells were suspended in 100 µL of FBS-free DMEM (Group 1 as control) or 100 µL of 75% PA-CM (Group 2) and supplemented with 100 µL Matrigel and, then, subcutaneously injected into the dorsum of nude mice, followed by injections of 200 µL FBS-free medium (Group 1) or 75% PA-CM (Group 2) at tumor sites every 3 days. Group 3, mice were injected with 2 × 10^6^ KKU213B cells in 100 µL FBS-free DMEM supplemented with 100 µL Matrigel; then, they were injected intraperitoneally with cisplatin (Kemoplat^®^, Vijayawada, India) (2 mg/kg body weight) every 3 days [22,23]. A group of mice was subcutaneously injected with only 2 × 10^6^ hPAMSCs in 200 µL of FBS-free medium supplemented with 100 µL Matrigel to determine whether hMSC transplantation alone generated tumors or had any negative effect in mice. Every 3–4 days after the initial treatment, the body weight of the nude mice was monitored and the tumor size was measured with a caliper. Tumor volume was calculated according to a standard formula: tumor volume = length × width^2^ × 0.52. Four weeks after injection, the mice in each group were euthanized for subsequent analysis. The tumor mass was harvested for further examination after the animals were terminated with 5% isoflurane anesthetization. The death of each animal was confirmed by a veterinarian.

### 2.11. Immunohistochemical Staining (IHC)

The resected tumor was fixed in 4% paraformaldehyde for embedding in paraffin. Sections of 4 μm thickness were cut from representative paraffin blocks and stained with hematoxylin and eosin (H&E), using standard protocols, or immunohistochemistry was performed. The tissue sections were deparaffinized with xylene and dehydrated using a graded concentration series of ethanol. They were then boiled in 10 mM sodium citrate buffer for 10 min in a microwave oven to recover antigens. Subsequently, the slides were incubated with 3% hydrogen peroxide in methanol for 30 min, blocked in 4% bovine serum albumin (BSA) for 1 h, and then incubated with the primary anti-cleaved caspase 3 antibody (1:300, Cell Signaling, USA) overnight at 4 °C. After incubation, the slides were incubated with mouse anti-rabbit IgG (1:500, Jackson ImmunoResearch Laboratories, USA) at room temperature for 1 h. Immunoreactivity was visualized using the NovaRED substrate kit (Vector, Torrance, CA, USA) and counterstained with hematoxylin. Immunohistochemical staining for Ki-67 was performed by validated automated staining on the Dako Omnis platform (Agilent Technologies, Carpinteria, CA, USA) with the primary anti-Ki-67 antibody Clone MIB-1 (1:300, GA626, Dako Omnis), followed by visualization with the DAKO OMNIS Flex HRP detection system. The slides were counterstained with hematoxylin, dehydrated with a graded concentration series of ethanol, and had a cover slip placed on them [24]. IHC staining was evaluated in 10 selected non-overlapping 400× magnification high-power field (HPF) areas using a light microscope. The stained sections were independently evaluated by expert pathologists blinded to initial evaluations.

### 2.12. Statistical Analysis

Data were expressed as the mean ± standard error of the mean (SEM). Statistical analyses were performed with GraphPad Prism version 8.0.2 (GraphPad Software, Inc., Boston, MA, USA). Comparisons between multiple groups were analyzed statistically by a one-way or two-way analysis of variance with the Tukey post hoc test. Differences were considered statistically significant at * *p* < 0.05, ** *p* < 0.01, and *** *p* < 0.001.

## 3. Results

### 3.1. Characterization of Human Placental Amniotic Membrane Mesenchymal Stromal Cells (hPAMSCs)

**Figure 1:**
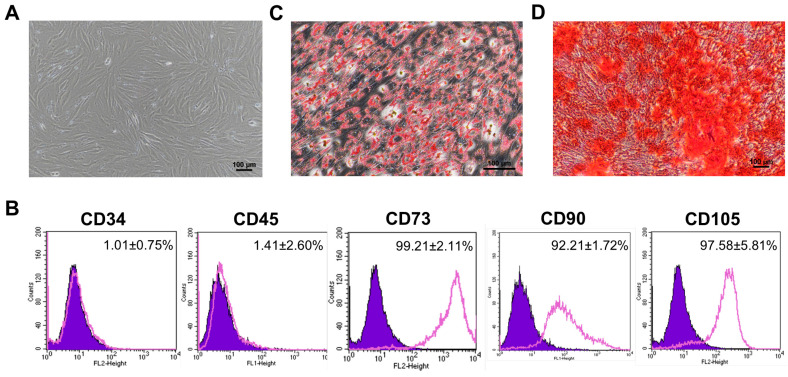
Characterization of human placental amniotic membrane mesenchymal stromal cells (hPAMSCs). (**A**) Fibroblast-like morphology of isolated hPAMSCs. (**B**) The flow cytometric analysis of the hPAMSCs showed positive for CD73, CD90, and CD105 and negative for CD34, and CD45 while the IgG isotype was used as a negative control (painted purple). (**C**) Potential of hPAMSCs for adipogenic differentiation demonstrated by the accumulation of lipid droplets stained with oil-red O staining (200× magnification). (**D**) Potential of hPAMSCs for osteogenic differentiation demonstrated by the deposition of calcium stained with alizarin-red S staining (100× magnification).

The hPAMSCs showed fibroblast-like morphologies (Figure 1A); homogenously expressed typical hMSC markers, CD73 (99.21 ± 2.11%), CD90 (92.21 ± 1.72%), and CD105 (97.58 ± 5.81%); and did not express hematopoietic markers, CD34 (1.01 ± 0.75%) and CD45 (1.41 ± 2.60%) (Figure 1B). Furthermore, these cells also differentiated into adipocytes (Figure 1C) and osteoblasts (Figure 1D) when cultured under appropriate conditions. These results suggest that the hPAMSCs established in this study exhibited the typical characteristics of hMSCs. 

### 3.2. PA-CM Suppresses CCA Cell Growth

**Figure 2:**
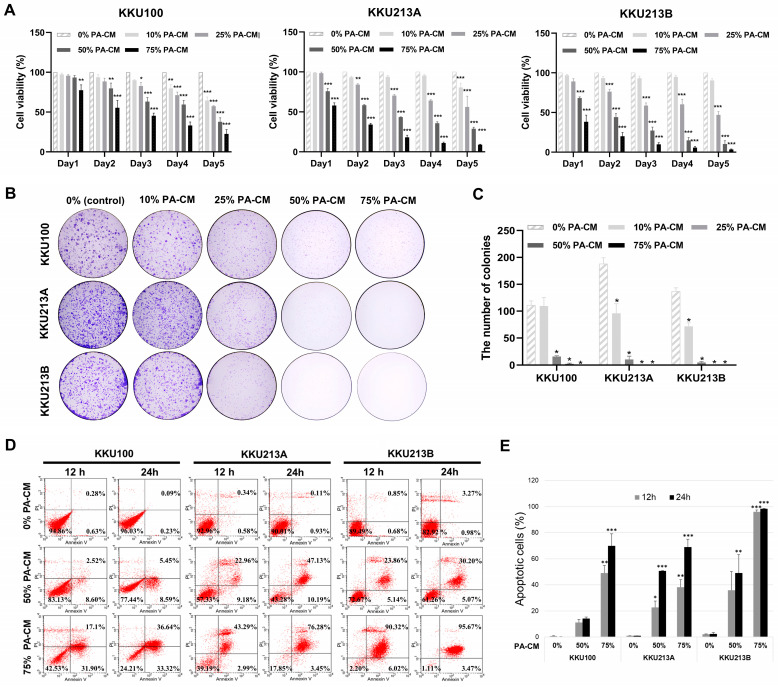
PA-CM suppresses cell growth and promotes apoptosis of CCA cell lines. The CCA cell lines, KKU100, KKU213A, and KKU213B, were cultured with different concentrations of PA-CM, including 0% (control), 10%, 25%, 50%, and 75%. (**A**) MTT assays showed that PA-CM significantly decreased the percentage of cell viability in three CCA cells in a dose- and time-dependent manner. (**B**,**C**) PA-CM significantly decreased the number of colony formations of three CCA cells. (**D**,**E**) PA-CM at 50% and 75% concentrations significantly increased the number of apoptotic cells in three CCA cells compared to the control. All data were presented as the mean ± SEM of three independent experiments. * *p* < 0.05, ** *p* < 0.01, and *** *p* < 0.001 vs. control.

To determine the effect of soluble factors derived from hPAMSCs on CCA cell growth, a conditioned medium derived from hPAMSCs (PA-CM) over a range of concentrations was used to culture three human CCA cell lines for 5 days. Results of the MTT assays showed that the proliferation of all three human CCA cell lines was significantly inhibited by PA-CM in a dose- and time-dependent manner compared to the control (Figure 2A). The reduction in relative CCA cell growth after being treated with 50% and 75% PA-CM was observed as early as 24 h compared to the control (93.41% and 77.40%, respectively, for KKU100; 75.78% and 57.91%, respectively, for KKU213A; and 68.22% and 38.27%, respectively, for KKU312B; Figure 2A). Consistent with this, PA-CM also significantly reduced the number of colony formations in all CCA cell lines compared to the control (0% PA-CM) in a dose-dependent manner (*p* < 0.001; Figure 2B,C). The inhibitory effect on the CCA cell proliferation and colony-forming capacity was clearly observed when the concentration of PA-CM was greater than 25%. These results suggest that soluble factors derived from hPAMSCs inhibited the proliferation and colony-forming capacity of human CCA cells in a dose- and time-dependent manner.

### 3.3. PA-CM Promotes CCA Cell Apoptosis

Three CCA cell lines were treated with 50% and 75% PA-CM for 24 h to investigate the effect of soluble factors derived from hPAMSCs on CCA cell apoptosis. Flow cytometry analysis showed that PA-CM dramatically decreased the percentages of viable CCA cells (annexin V−/PI−) and increased the percentages of apoptotic CCA cells, which included both early (annexin V+/PI−) and late (annexin V+/PI+) stages of apoptosis, compared to controls, in a dose- and time-dependent manner. The reduction in CCA cell viability was observed as early as 12 h compared to the controls and was clearly observed when the concentration of PA-CM was above 50% of the control (Figure 2D,E). After being treated with 75% PA-CM for 24 h, the percentages of viable CCA cells decreased from 96.03% to 24.21% for KKU100, from 90.01% to 17.85% for KKU213A, and from 82.92% to 1.11% for KKU213B, compared to the controls (0% PA-CM) (Figure 2D). At the same time, the percentages of apoptotic CCA cells increased from 0.32% to 69.96% for KKU100, from 1.04% to 73.73% for KKU213A, and from 4.25% to 99.14% for KKU213B, compared to the controls (Figure 2E). The results suggested that CCA cell apoptosis can be promoted in a dose- and time-dependent manner by soluble factors derived from hPAMSCs.

### 3.4. PA-CM Induces the Loss of Mitochondrial Membrane Potential in CCA Cells

**Figure 3:**
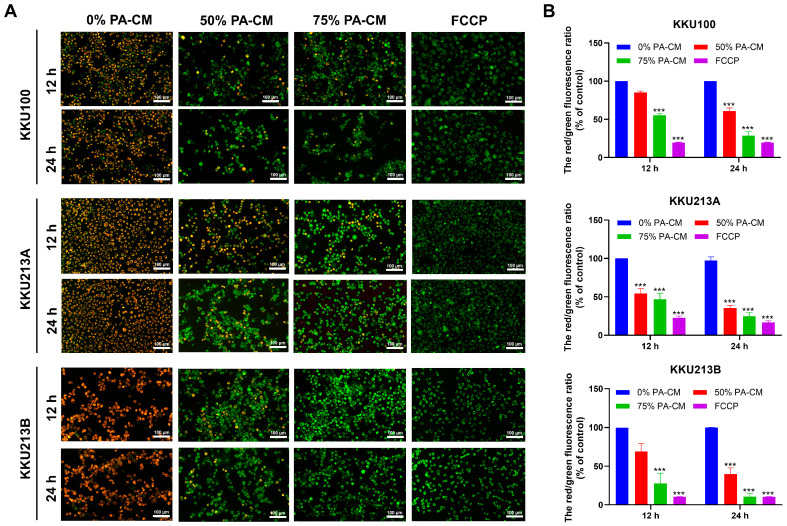
PA-CM induces the loss of mitochondrial membrane potential (∆Ψm) in CCA cell lines. (**A**,**B**) CCA cells (KKU100, KKU213A, and KKU213B) were treated with 0%, 50%, and 75% concentrations of PA-CM for 12 and 24 h; then, they were stained with JC-1 dye. FCCP was used as a positive control. (**A**) All CCA cells treated with PA-CM exhibited a dose- and time-dependent increase in green fluorescence (mitochondrial depolarization) and a decrease in red fluorescence (normal mitochondria), as observed by fluorescence microscopy. (**B**) Using a microplate reader, quantitative analysis of JC-1 staining showed that the red/green fluorescence ratio significantly decreased in all CCA cells after culture with PA-CM in a dose-dependent manner. This indicates that PA-CM induced the loss of mitochondrial membrane potential in CCA cells. Results are shown as the mean ± SEM of three independent experiments. Scale bar = 100 μm. *** *p* < 0.001 vs. control.

Loss of mitochondrial membrane potential or mitochondrial depolarization is one of the defining characteristics of apoptosis. JC-1 staining was performed to determine the effect of soluble factors derived from hPAMSCs on the mitochondrial membrane potential of CCA cells. The results revealed that PA-CM increased mitochondrial depolarization (indicated by green fluorescence) relative to normal mitochondria (indicated by red fluorescence) compared to the control (Figure 3A). As with its effect on CCA cell apoptosis, quantitative analysis of JC-1 staining showed that after 24 h of treating CCA cell lines with 50% and 75% PA-CM, the red/green fluorescence ratio for KKU100 was reduced to 60.67% and 28.59%, respectively; 35.50% and 24.52%, respectively, for KKU213A; and 39.83% and 10.61%, respectively, for KKU213B, compared to the controls (0% PA-CM) (Figure 3B). The results indicated that PA-CM significantly increased mitochondrial depolarization in all three human CCA cells, compared to the control, in a dose- and time-dependent manner.

### 3.5. PA-CM Induces CCA Cell Apoptosis through the Mitochondrial-Mediated Caspase Pathway

**Figure 4:**
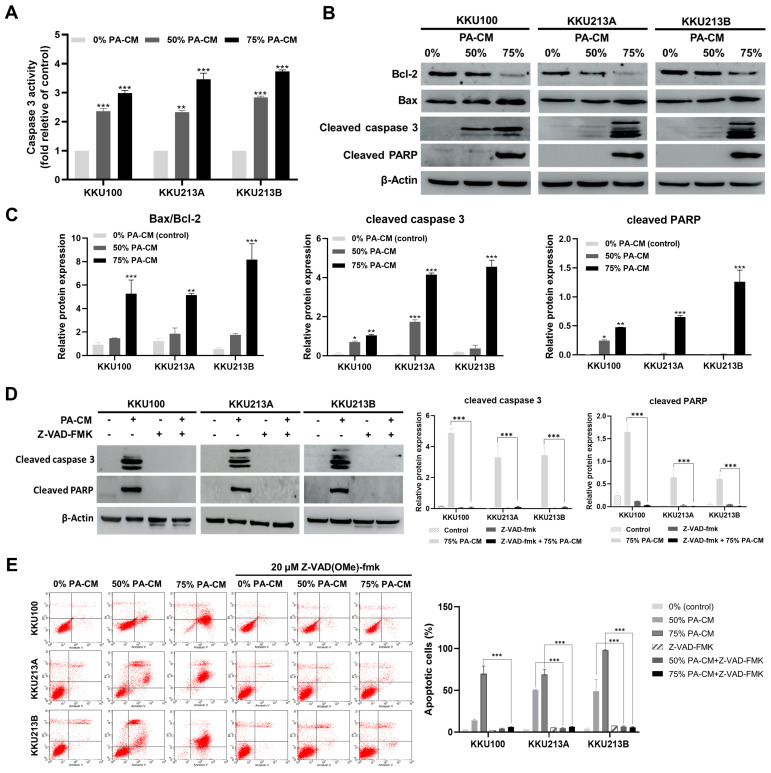
PA-CM induces apoptosis through mitochondrial-mediated caspase in CCA cell lines. CCA cells (KKU100, KKU213A, and KKU213B) were cultured with PA-CM (0%, 50%, and 75%) for 8 h. (**A**) PA-CM significantly increased caspase 3 activity in three CCA cells compared to the control. (**B**) PA-CM decreased Bcl-2 expression and increased Bax, cleaved caspase 3, and cleaved PARP expression levels in three CCA cells. (**C**) The relative expression of the Bax/Bcl-2 ratio, cleaved caspase 3, and cleaved PARP proteins increased significantly when CCA cell lines were cultured with PA-CM, compared to the control. (**D**) CCA cells were pretreated with Z-VAD(OMe)-FMK (pan-caspase inhibitor) for 1 h and were then treated with PA-CM (0% and 75%) for 8 h. Western Blot results showed that pre-treatment of CCA cells with Z-VAD(OMe)-FMK significantly decreased the expression levels of cleaved caspase 3 and cleaved PARP (*p* < 0.001). (**E**) CCA cells were pretreated with Z-VAD(OMe)-FMK for 1 h; then, they were cultured with PA-CM (0%, 50%, and 75%) for 24 h. Flow cytometric analysis with annexin V–PI staining showed a decrease in CCA cell apoptosis after Z-VAD(OMe)-FMK treatment. All data are presented as the mean ± SEM of three independent experiments. * *p* < 0.05, ** *p* < 0.01, and *** *p* < 0.001.

To examine if PA-CM induced CCA apoptosis via the caspase pathway, CCA cells were treated with 50% and 75% PA-CM for 8 h, whereupon caspase 3 activity was determined. The results showed that the level of caspase 3 activity in all three human CCA cell lines significantly increased following PA-CM treatment, compared to the control, in a dose-dependent manner (Figure 4A). PA-CM also reduced, in a dose-dependent manner, the level of Bcl-2, an anti-apoptotic protein, while increasing the levels of several pro-apoptotic proteins, including Bax and cleaved caspase 3, compared to controls (Figure 4B,C and Figure S1). Moreover, when all CCA cell lines were cultured with 75% PA-CM, the expression of cleaved PARP increased significantly compared to the control (Figure 4B,C and Figure S1). Furthermore, 75% PA-CM did not increase the levels of cleaved caspase 3 and cleaved PARP in the presence of Z-VAD(OMe)-FMK, a pan-caspase inhibitor (Figure 4D and Figure S2). The addition of Z-VAD(OMe)-FMK also abolished the pro-apoptotic effect of 50% and 75% PA-CM in the three human CCA cells (Figure 4E), thus confirming that PA-CM promoted CCA cell apoptosis by activating a caspase-dependent pathway that could lead to a loss of mitochondrial membrane potential in these cells.

### 3.6. PA-CM Suppresses the IL-6-Mediated JAK2/STAT3 Signaling Pathway in CCA Cells

**Figure 5:**
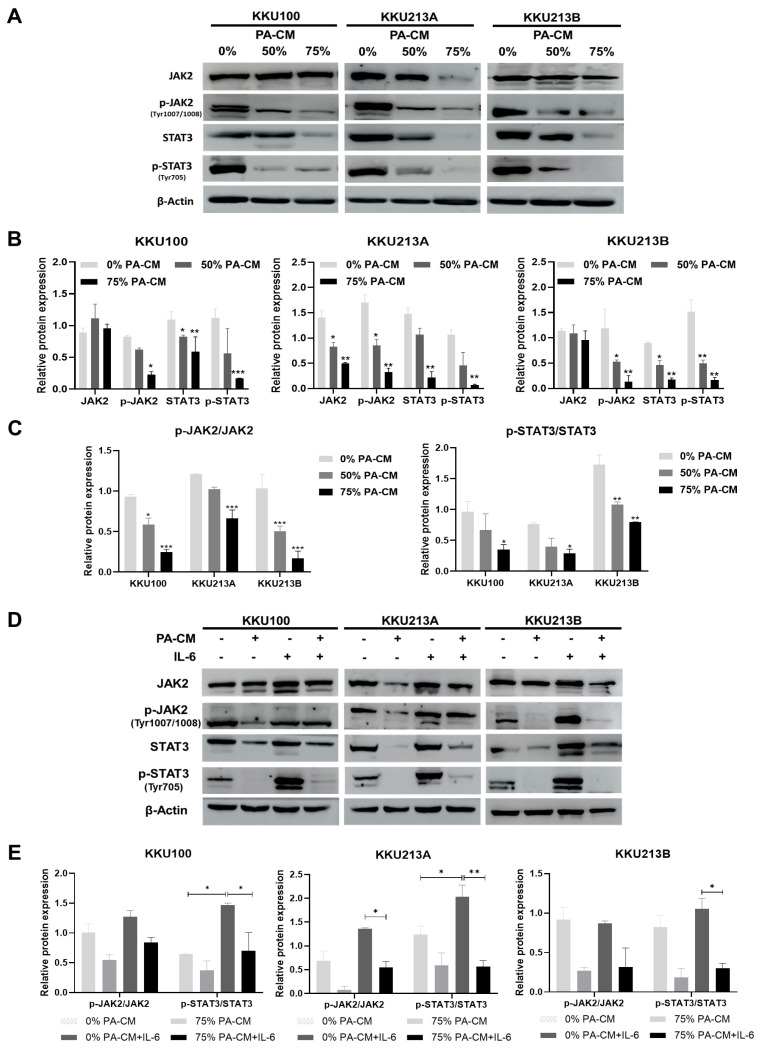
PA-CM inhibits IL-6-induced JAK2/STAT3 activation in CCA cell lines. (**A**–**C**) CCA cells (KKU100, KKU213A, and KKU213B) were cultured with PA-CM (0%, 50%, and 75%) for 8 h. Western Blot analysis showed that PA-CM decreased the expression of total and phosphorylated JAK2 and STAT3 in all CCA cells in a dose-dependent manner. (**D**,**E**) CCA cells were pretreated with 75% PA-CM for 8 h; then, they were stimulated with IL-6 (100 ng/mL) for 30 min. Western Blot analysis showed that 75% PA-CM reduced the p-JAK2/JAK2 and p-STAT3/STAT3 ratios in all CCA cells, compared to the control (0% PA-CM). The addition of IL-6 also increased the ratio of p-JAK2/JAK2 in KKU100 and KKU213B cells and increased the ratio of p-STAT3/STAT3 in all CCA cells. These results demonstrated that PA-CM inhibited IL-6-induced JAK2/STAT3 signaling in all CCA cells. * *p* < 0.05, ** *p* < 0.01, and *** *p* < 0.001.

To determine whether PA-CM induced the apoptosis of CCA cell lines through the JAK2/STAT3 signaling pathway (which has been shown to play a critical role in CCA survival), the levels of phosphorylated JAK2 and STAT3 proteins in PA-CM-treated CCA cells were determined by Western Blotting. The results revealed that PA-CM significantly decreased the levels of phosphorylated JAK2 (Y1007/Y1008) and phosphorylated STAT3 (tyr705) proteins in three human CCA cells in a dose-dependent manner (Figure 5A–C and Figure S3). Because IL-6 is a critical cytokine known to promote CCA progression by activating JAK2/STAT3 signaling, we investigated whether PA-CM inhibited IL-6-induced JAK2/STAT3 activation in CCA cells. Three CCA cells were pretreated with 75% PA-CM for 8 h and then treated with 100 ng/mL IL-6 for 30 min. The addition of IL-6 significantly increased the levels of p-JAK2, JAK2, p-STAT3, and STAT3 in all these CCA cells (Figure 5D,E). As shown in Figure 5D,E and Figure S4, the levels of phosphorylated JAK2 and phosphorylated STAT3 in CCA cells pretreated with 75% PA-CM prior to IL-6 treatment decreased compared to CCA cells treated with IL-6 alone. Likewise, the ratios of p-JAK2/JAK2 and p-STAT3/STAT3 in CCA cells pretreated with 75% PA-CM prior to IL-6 treatment decreased significantly compared to CCA cells treated with IL-6 alone (Figure 5E), indicating that PA-CM prevents the IL-6-induced JAK2/STAT3 signaling pathway in CCA cells. Taken together, we conclude that PA-CM suppressed JAK2/STAT3 signaling in human CCA cells and that its suppressive effect might be mediated, at least in part, by inhibiting IL-6.

### 3.7. PA-CM Suppresses CCA Cell Growth In Vivo by Inducing Apoptosis

**Figure 6:**
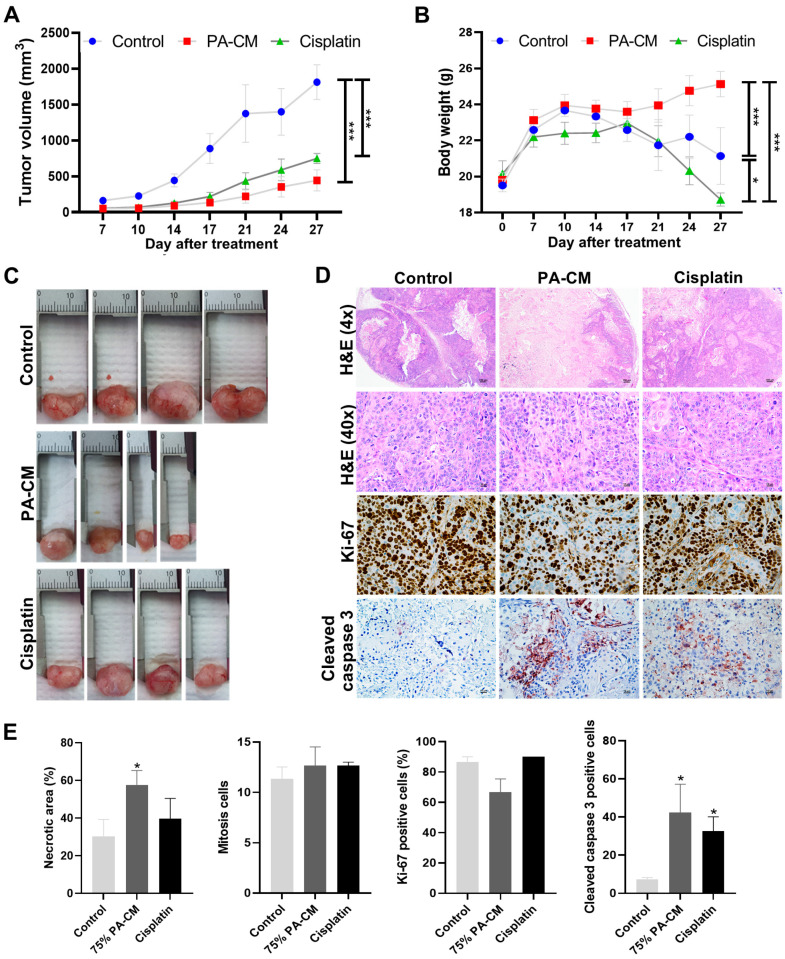
PA-CM suppresses CCA growth in mice by inducing apoptosis. BALB/c-nu/nu mice (*n* = 4/group) with subcutaneous KKU213B tumor xenografts treated with D-MEM (control), 75% PA-CM, or cisplatin. (**A**) The tumor volume and (**B**) body weight of all mice were determined every 4 days. (**C**) On day 27 after treatment, the tumor mass was isolated. (**D**) Representative of H&E staining and IHC analysis for Ki-67 and cleaved caspase 3 (×400 magnification) in the tumor section of mice. (**E**) Quantitative analysis of necrotic area (%), amount of mitosis (cells/high-power field), Ki-67 positive cells (%), and number of cleaved caspase 3 positive cells in xenograft tissues from different groups (* *p* < 0.05 vs. control). Data were presented as the mean ± SEM. * *p* < 0.05, *** *p* < 0.001 (*n* = 4).

To evaluate the effects of PA-CM on CCA cell growth in vivo, BALB/c-nu/nu mice were transplanted with KKU213B cells to establish CCA xenografts. After transplantation, tumor size was measured every four days. We found that tumor volume increased rapidly in the untreated group (0% PA-CM) but increased noticeably more slowly in mice treated with 75% PA-CM or the chemotherapeutic agent cisplatin. As expected, mice transplanted with hPAMSCs alone did not develop tumors and tumor volume in the transplanted hPAMSCs were undetectable 14 days after transplantation (Figure S5). Tumor volumes in the two treated groups (PA-CM and cisplatin) were significantly lower than in the untreated group (1811.77 ± 241.76 mm^3^) (Figure 6A). Among the two treatment groups, PA-CM-treated mice had lower tumor volumes (442.78 ± 146.92 mm^3^) than those of the cisplatin-treated group (747.97 ± 70.12 mm^3^) (Figure 6A). The average body weight of the mice was also measured every four days and used as an additional indicator of health. Consistent with the results for tumor volume, the weights of PA-CM-treated mice were significantly higher than those of their cisplatin-treated and untreated counterparts (Figure 6B). After treatment for 7 and 27 days, the average body weight of the mice decreased from 22.19 ± 0.56 g to 18.72 ± 0.36 g for the cisplatin-treated group and from 22.59 ± 0.29 g to 21.14 ± 1.57 g for the untreated group; meanwhile, the average body weight of the mice treated with PA-CM increased from 23.12 ± 0.60 g to 25.13 ± 0.70 g. Although cisplatin treatment reduced tumor volume, it did not increase the average body weight of the mice compared to the untreated group (Figure 6B).

On the 27th day after transplantation, all mice were sacrificed and the tumors were harvested. Tumor sizes in the 75% PA-CM-treated group were much smaller than in other groups (Figure 6C). Although the tumor size in the cisplatin-treated groups (12.01 ± 0.33 mm) was smaller than in the untreated groups (17.28 ± 0.67 mm), these tumors were larger than those of the PA-CM-treated group (9.43 ± 1.08 mm) (Figure 6C). Consistent with this, H&E staining and immunohistochemical analysis showed that the tumors of the PA-CM-treated group had a significantly higher percentage of necrotic areas and contained a higher number of apoptotic cells with a higher level of cleaved caspase 3 compared to the untreated control (Figure 6D, *p* < 0.05). Although the tumors in the cisplatin-treated group contained significantly higher numbers of apoptotic cells with increased cleaved caspase 3 levels compared to the untreated controls, their tumors did not contain a significantly higher percentage of necrotic areas compared to the controls (Figure 6D,E). Unlike the number of apoptotic cells, the number of mitotic and Ki-67 positive tumor cells in the two treated groups was not significantly different from their untreated counterparts (Figure 6D). Taken together, our results showed that PA-CM decreased the volume of CCA tumors in vivo by promoting the apoptosis of these cells. 

## 4. Discussion

According to previous studies, mesenchymal stromal cells (MSCs) secrete a variety of cytokines that, depending on the circumstances, stimulate [25,26,27] or inhibit [28,29] the progression of various cancers. Although the effects of MSCs on several solid tumors have been extensively explored, their role in CCA growth and progression remains controversial. The soluble factors released from MSCs have been shown to affect the biological properties of cancer cells [30] and are believed to be the key mechanism underlying their therapeutic effects. Therefore, the present study aims to evaluate the effects of soluble factors in hPAMSC-derived conditioned medium (PA-CM) on the growth and apoptosis of human CCA cells (KKU100, KKU213A, and KKU213B), using in vitro and in vivo models. 

We found that PA-CM inhibited proliferation and increased apoptosis in all three human CCA cells in a dose- and time-dependent manner. These results are consistent with several previous reports showing that hMSCs suppress proliferation and promote apoptosis in various types of cancer cells, including gastric cancer, breast cancer, and glioma [31,32,33,34]. Dysregulation of apoptotic signaling promotes cancer progression by increasing the survival, drug resistance, and relapse of cancer cells [35]; thus, the induction of apoptosis is considered an effective cancer treatment. The balance of Bax (pro-apoptotic protein) and Bcl-2 (anti-apoptotic protein) regulates the integrity of the mitochondrial membrane [36]. An increased Bax/Bcl-2 ratio increases the permeability of the mitochondrial outer membrane, resulting in the release of cytochrome C, which, in combination with Apaf-1 and procaspase 9 [37], activates the apoptosis pathway by activating caspase 3 and PARP [38,39].

We found that PA-CM induced the loss of mitochondrial membrane potential (∆Ψm) in CCA cells by increasing the Bax/Bcl-2 ratio and up-regulating the levels of cleaved caspase 3 and cleaved PARP proteins in these cells. Furthermore, the addition of the caspase inhibitor z-VAD(OMe)-FMK completely abolished the apoptotic-inducing effect of PA-CM in CCA cells, confirming that the pro-apoptotic effect of PA-CM was mediated through a caspase-dependent pathway. These findings are consistent with previous research showing that human endometrial-derived MSCs exhibit antitumor properties in human epithelial ovarian cancer cells by altering mitochondrial membrane potential and promoting apoptosis in these cells [40].

To understand the mechanism by which PA-CM induced apoptosis in CCA cells, we focused on JAK2/STAT3 signaling, which plays an important role in the growth and survival of many cancers, including CCA [41,42,43,44,45,46]. Furthermore, overactivation of JAK2/STAT3 signaling triggered by elevated levels of IL-6 has been shown to increase the expression levels of several anti-apoptotic genes, such as *BCL2*, *BCL-XL*, *BIRC5*, and *CCN* in CCA cells [47,48]. Our results showed that PA-CM suppressed STAT3 activation in CCA cells by inhibiting the phosphorylation of JAK2, which is a key tyrosine kinase in the regulation of STAT3 activation. Furthermore, PA-CM also significantly inhibited IL-6-induced JAK2/STAT3 activation in CCA cells. The inhibition of JAK2/STAT3 signaling by PA-CM could then lead to an increase in the level of apoptotic CCA cells observed in this study. The results, herein, confirm those in our previous study, which showed that soluble factors released from chorion-derived hMSCs inhibited JAK2/STAT3 signaling, reduced Bcl-2 expression, and increased Bax expression in CCA cells [49].

Consistent with our in vitro study, our in vivo study using a xenografted mouse model showed that PA-CM significantly reduced CCA growth and prevented tumor-induced weight loss in these mice. Although cisplatin also reduced CCA growth compared to the untreated group, it failed to prevent tumor-induced weight loss in these mice. These results suggest that PA-CM is more effective than cisplatin in suppressing CCA growth in vivo, resulting in better health in mice. IHC analysis also revealed that PA-CM inhibited CCA growth by triggering apoptosis, as demonstrated by an increase in the level of cleaved caspase 3 in these cells. These findings are consistent with a previous study, which found that amniotic-derived hMSCs induce hepatocellular carcinoma cell apoptosis in the mouse xenograft model [50]. 

## 5. Conclusions

In summary, our results collectively suggest that PA-CM induces apoptosis in CCA cells by releasing soluble factors that inhibit JAK2/STAT3 signaling and, similarly, inhibit the expression of the Bcl-2 anti-apoptotic protein. The decline in the Bcl-2 level, which results in an increase in the BAX/Bcl-2 ratio, is correlated with an increase in the level of CCA cell apoptosis and could, therefore, be responsible for CCA cell apoptosis. Furthermore, the suppressive effect of PA-CM on CCA in vivo is greater than that of cisplatin. We believe that PA-CM could be used as an alternative therapy or in combination with a conventional chemotherapeutic drug to improve the therapeutic efficiency of CCA treatment and improve the quality of life of CCA patients.

## Data Availability

The datasets generated during and/or analyzed during the current study are available from the corresponding author on reasonable request.

## Supplementary Materials

The following supporting information can be downloaded at: https://www.mdpi.com/article/10.3390/cells12242788/s1, Figure S1: Full-length blots for Figure 4B; Figure S2: Full-length blots for Figure 4D; Figure S3: Full-length blots for Figure 5A; Figure S4: Full-length blots for Figure 5D; Figure S5: The tumor volume and the average body weight of the transplanted hPAMSC mice.
