# Supplementary material for: The Human Placental Amniotic Membrane Mesenchymal-Stromal-Cell-Derived Conditioned Medium Inhibits Growth and Promotes Apoptosis of Human Cholangiocarcinoma Cells In Vitro and In Vivo by Suppressing IL-6/JAK2/STAT3 Signaling
Source: Cells. 2023 Dec 7;12(24):2788. doi: 10.3390/cells12242788 (PMC10742162; doi:10.3390/cells12242788)
Supplement: Supplementary file 1 [file cells-12-02788-s001.zip › cells-2685983-supplementary.pdf]

Supplementary data

Repeat 1

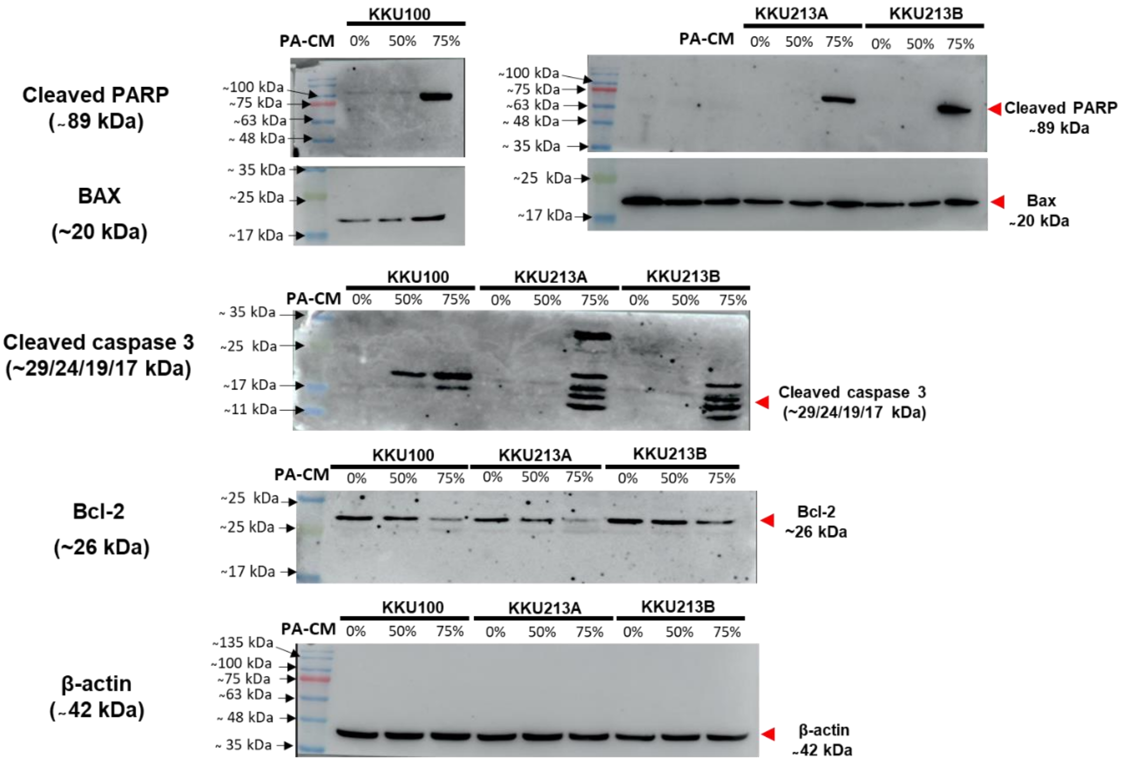

Repeat 2

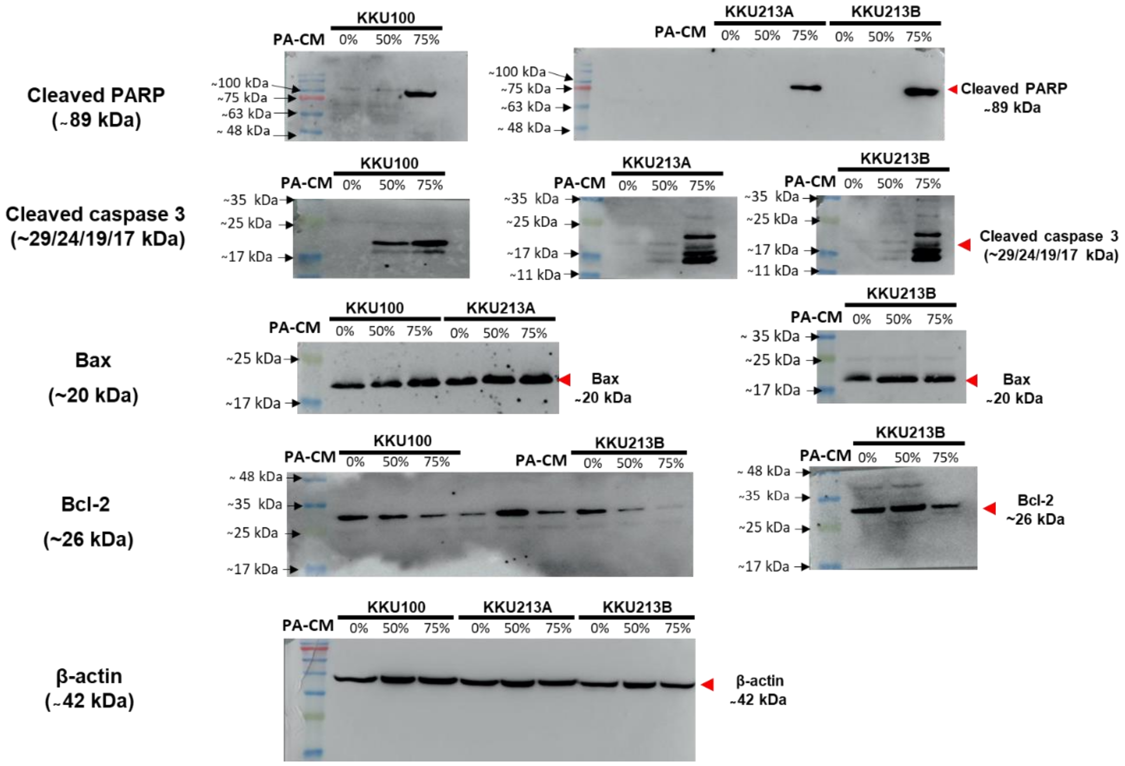

Repeat 3

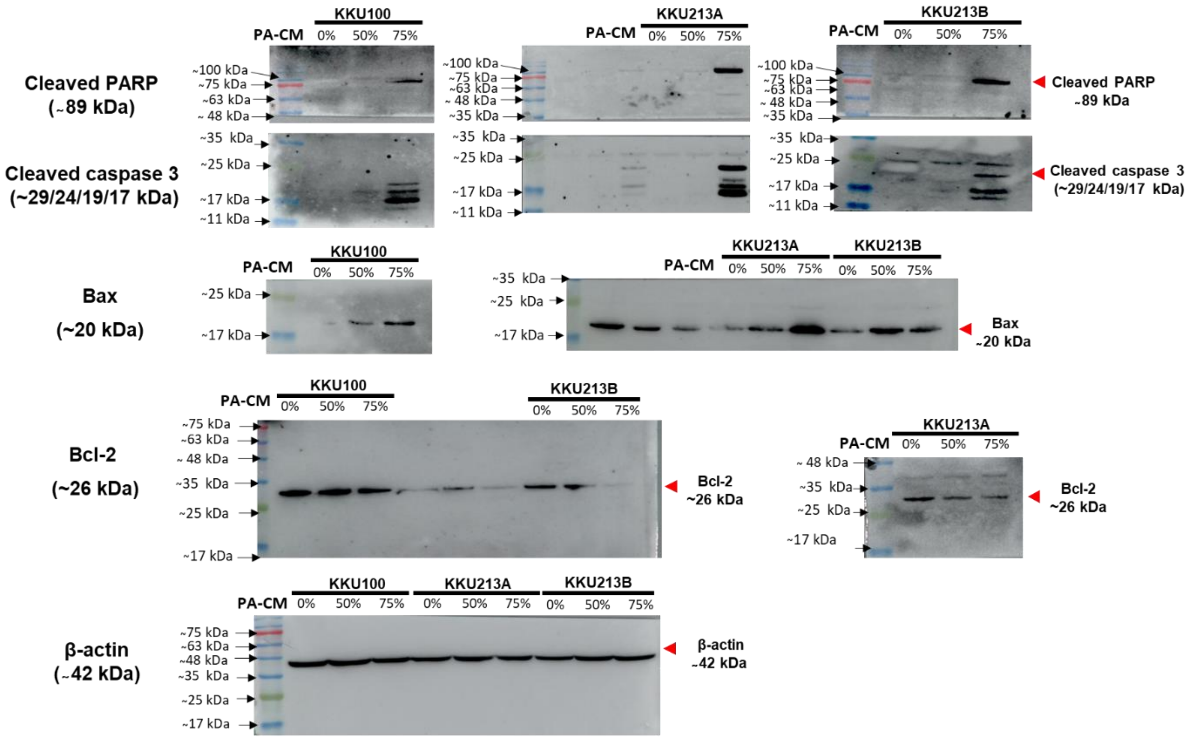

Figure S1. Full-length blots for Figure 4B. Three repeats of the immunoblotting for Cleaved PARP (89 kDa), β-actin (42 kDa), Cleaved caspase 3 (29/24/19/17 kDa), Bcl-2 (26 kDa) and Bax (20 kDa).

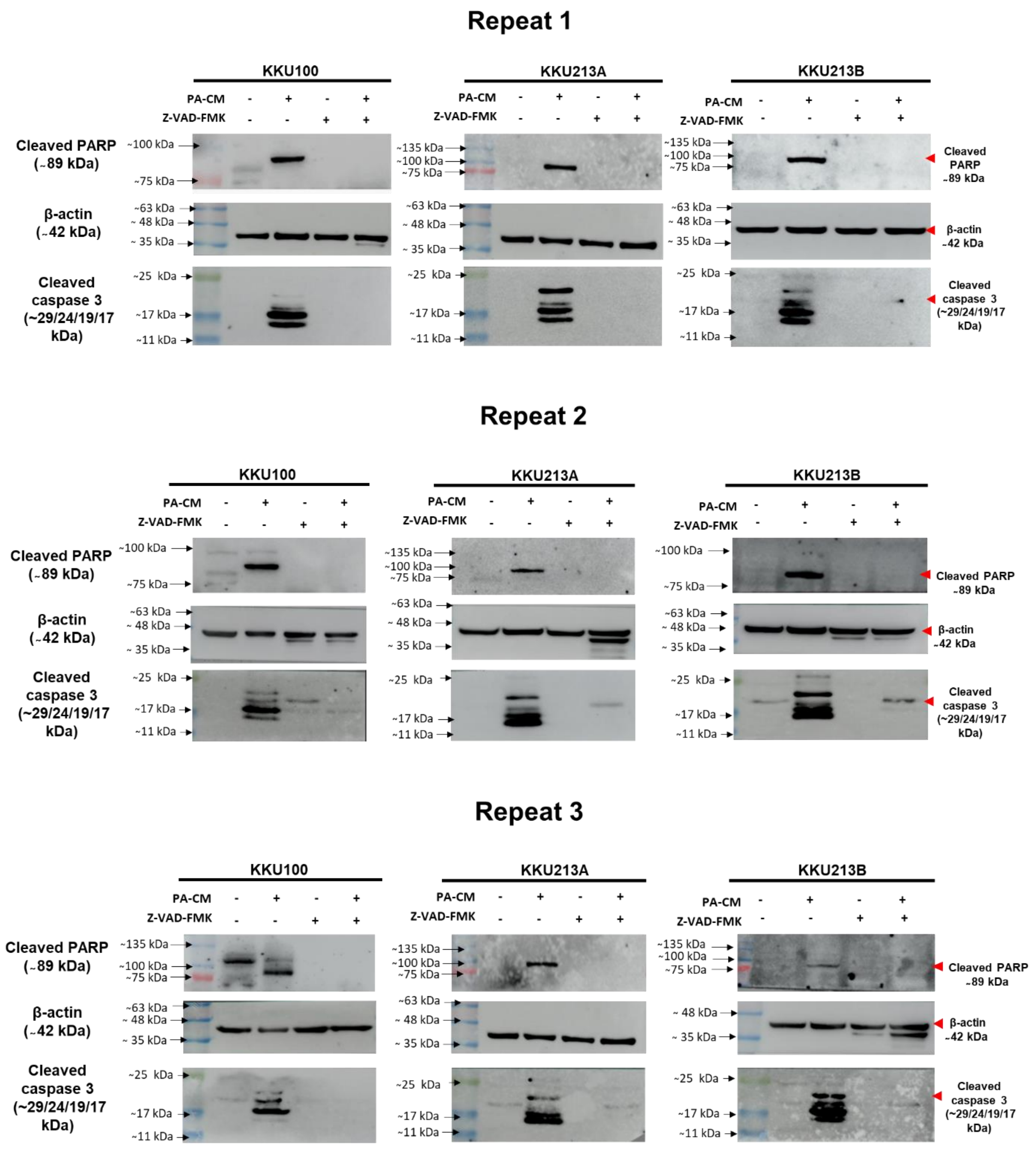

Figure S2. Full-length blots for Figure 4D. Three repeats of the immunoblotting for Cleaved PARP (89 kDa), β-actin (42 kDa) and Cleaved caspase 3 (29/24/19/17 kDa).

Repeat 1

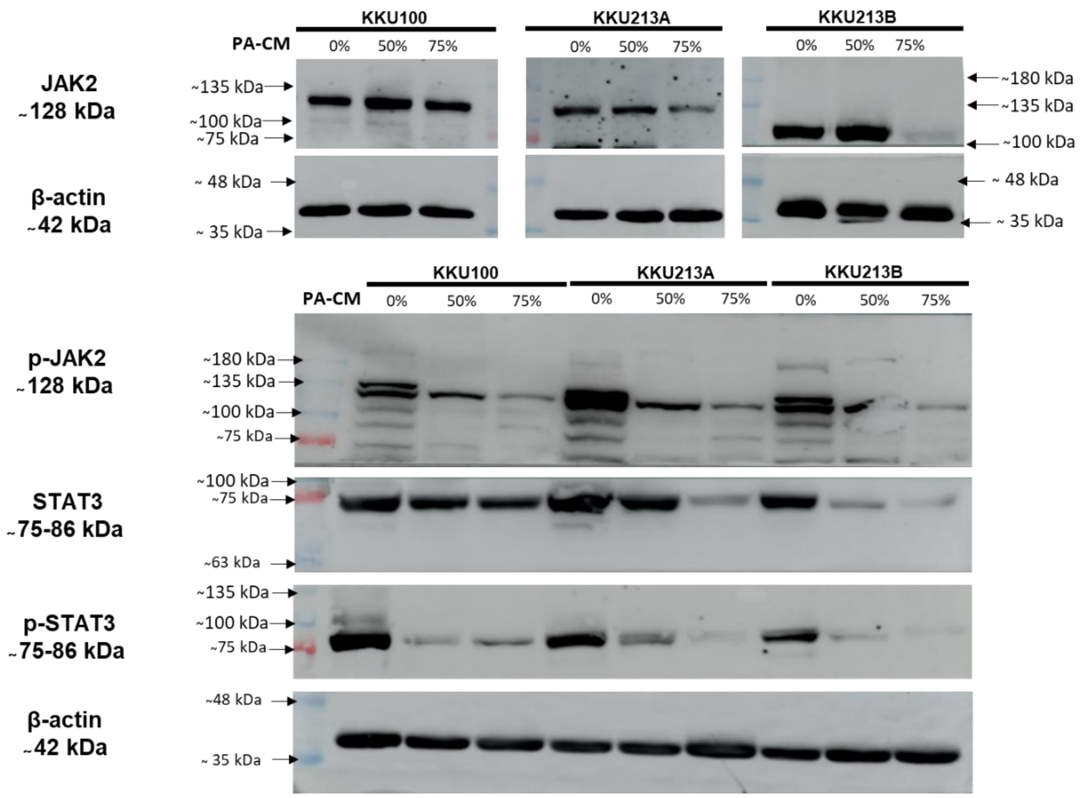

Repeat 2

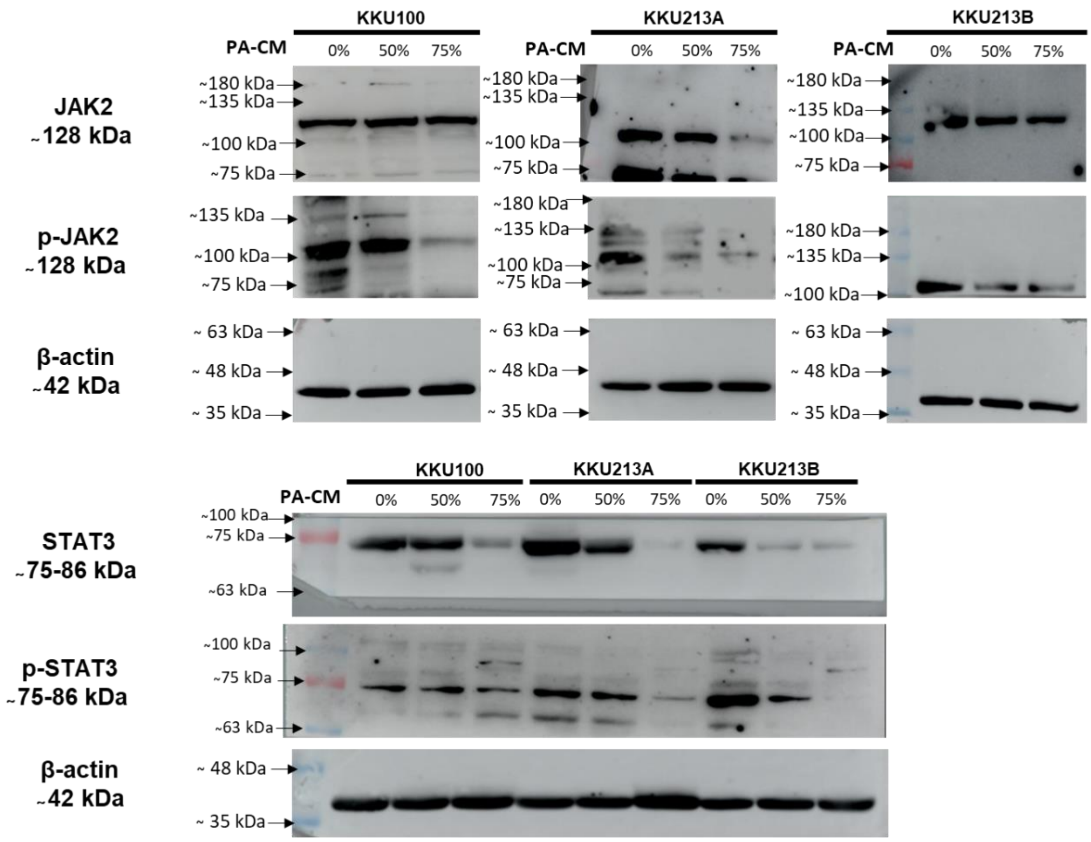

Repeat 3

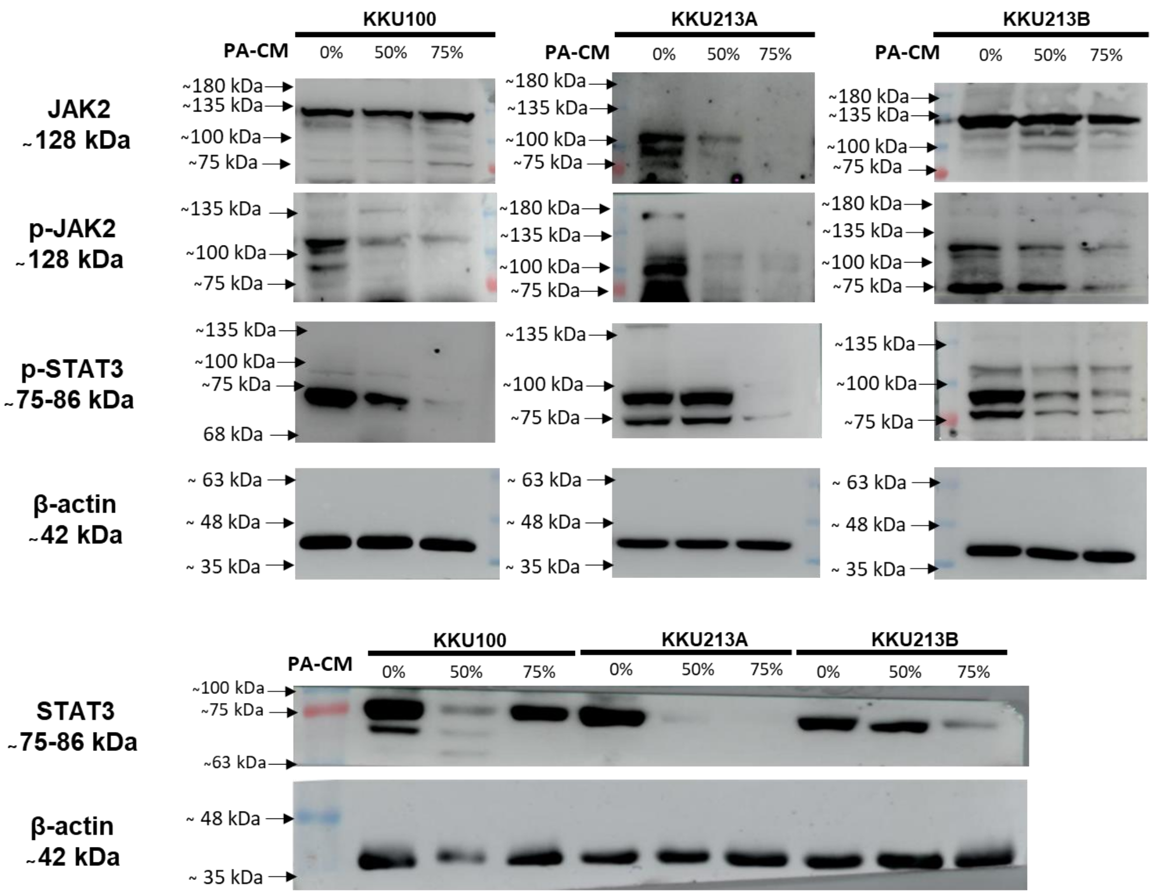

Figure S3. Full-length blots for Figure 5A. Three repeats of the immunoblotting for JAK2 (128 kDa), p-JAK2 (128 kDa), STAT3 (75-86 kDa), p-STAT3 (75-86 kDa) and β-actin (42 kDa).

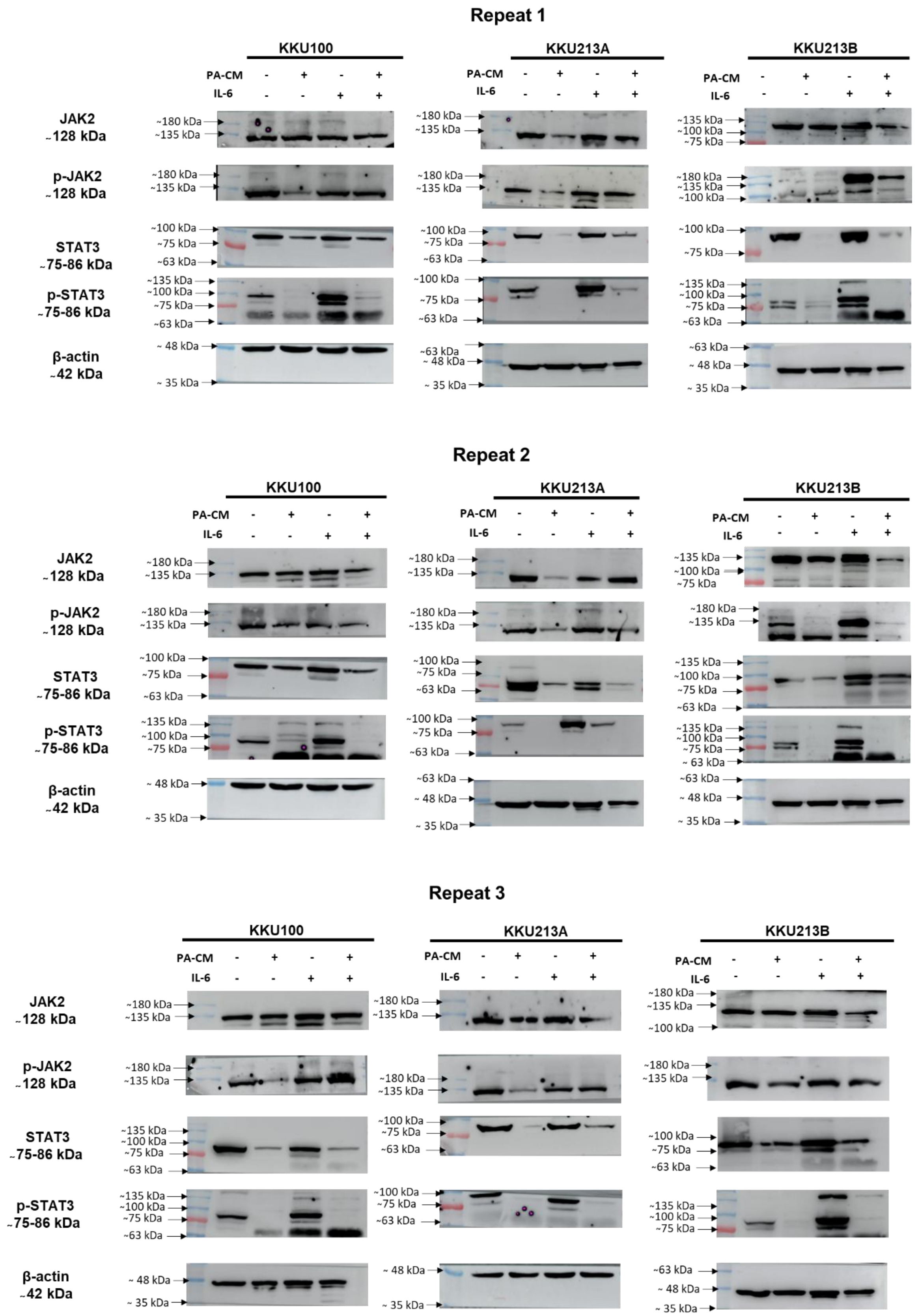

Figure S4. Full-length blots for Figure 5D. Three repeats of the immunoblotting for JAK2 (128 kDa), p-JAK2 (128 kDa), STAT3 (75-86 kDa), p-STAT3 (75-86 kDa) and β-actin (42 kDa).

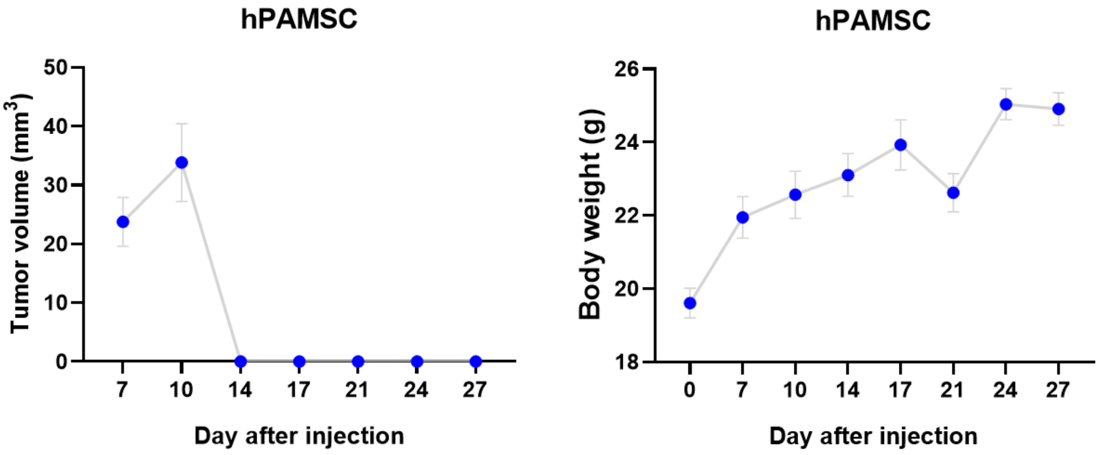

Figure S5. The tumor volume and the average body weight of the transplanted hPAMSC mice. Mice (n=4) were subcutaneously injected with 2×10^6 hPAMSC in 200 µL of FBS-free medium supplemented with 100 µL Matrigel. The tumor size was measured with a caliper, and the mice's body weight was monitored every 4 days after the initial injection. Fourteen days after transplantation, the transplanted hPAMSC was undetectable and had no effect on tumor-induced weight loss in these mice.
